# Molecular Surveillance, Evolution, and Vaccine Strain Match of the *HA* and *NA* Genes of 2009 H1N1 Pandemic Virus Circulating in Riyadh, Saudi Arabia

**DOI:** 10.3390/ijms27031412

**Published:** 2026-01-30

**Authors:** Reem M. Aljowaie, Ibrahim M. Aziz, Mohamed A. Farrag, Abdulaziz M. Almuqrin, Fahad N. Almajhdi

**Affiliations:** 1 Department of Botany and Microbiology, College of Science, King Saud University, P.O. Box 2455, Riyadh 11451, Saudi Arabia; iaziz@ksu.edu.sa (I.M.A.); mfarrag@ksu.edu.sa (M.A.F.); 2 Department of Clinical Laboratory Sciences, College of Applied Medical Sciences, King Saud University, P.O. Box 10219, Riyadh 12372, Saudi Arabia; aalmuqrin@ksu.edu.sa

**Keywords:** influenza virus, influenza surveillance, viral evolution, vaccine effectiveness, phylogenetic analysis

## Abstract

Influenza viruses are characterized by their high mutation rates which require continuous molecular surveillance to ensure the annual effectiveness of influenza vaccines. The current study aimed to investigate the molecular evolution and vaccine match of the 2009 pandemic (A(H1N1) pdm09) virus circulating in Riyadh, Saudi Arabia. A total of 380 nasopharyngeal aspirates (NPAs) were collected during the 2020–2023 winter seasons from patients with influenza-like illness. Influenza A virus (IAV) detection, typing, and amplification of *hemagglutinin (HA)* and *neuraminidase (NA)* genes were achieved using one-step RT-PCR. The full-length *HA* and *NA* genes of 14 selected A(H1N1) pdm09 isolates were sequenced and used for sequence and phylogenetic analysis, which also included sequences of seven A(H1N1) pdm09 isolates collected in Riyadh during the 2024–2025 season. IAV was detected in 17.11% samples; A/H3N2 (9.21%) was somewhat more prevalent than A(H1N1) pdm09 (7.89%). Children aged 0–4 years had the highest incidence rate of infection. Comparing the HA1 domain of A(H1N1) pdm09 isolates circulating in Riyadh to the current vaccine strains (A/Wisconsin/67/2022 and A/Victoria/4897/2022), a total of 24 amino acid substitutions were identified. O-linked and N-linked glycosylation sites in the HA and NA proteins of the Riyadh isolates coincided with those of the two vaccine strains. The receptor-binding domain (130-loop) of the HA1 domain showed a persistent S137P substitution in all study isolates; this mutation is not present in the current vaccination strain. This finding suggests a potential antigenic mismatch between the current vaccine and the circulating A(H1N1) pdm09 strains in Riyadh, warranting hemagglutination inhibition (HAI) assays to confirm the impact of the S137P substitution on antigenicity and immune evasion. As shown above, ongoing molecular surveillance is essential for guiding the yearly selection of vaccine candidates to increase efficacy.

## 1. Introduction

Human influenza A viruses (IAVs) significantly affect public health and the global economy by leading to seasonal epidemics and sporadic pandemics, which result in high morbidity and mortality rates each year [1]. As stated by the International Committee on Taxonomy of Viruses (ICTV), influenza viruses are classified under the *Orthomyxoviridae* family and are defined by an enveloped viral particle that contains a segmented genome made up of negative-sense, single-stranded RNA [2]. In this family, viruses are organized into four distinct types: influenza A, B, C, and D. IAV is the most frequently encountered type that circulates and triggers seasonal epidemics in humans, in addition to IBV [3,4].

In April 2009, a new strain of the influenza A(H1N1) virus, which caused the 2009 pandemic (A(H1N1) pdm09) virus, appeared concurrently in Mexico and the United States, swiftly disseminating across the globe and resulting in the first pandemic of the 21st century [5,6]. This virus resulted from several reassortment events that involved viruses from swine, birds, and humans. By the end of the pandemic period, the A(H1N1) pdm09 strain had caused over 18,000 fatalities globally [7,8]. In Saudi Arabia, by December 2009, there were 15,850 laboratory-confirmed cases and 124 recorded fatalities from the illness [9].

During the coronavirus disease 2019 (COVID-19) pandemic, non-pharmaceutical interventions (such as lockdowns, mask-wearing, and social distancing) led to a substantial global decline in seasonal influenza activity. In Europe, the European Centre for Disease Prevention and Control (ECDC) reported very low influenza circulation during the 2020–2021 season (with detections near inter-seasonal levels), followed by a return of activity in 2021–2022 (though late-onset and shorter than pre-pandemic seasons) and near pre-pandemic levels by 2022–2023 [10,11]. This pattern reflects reduced viral transmission during peak COVID-19 restrictions, with potential implications for influenza lineage dynamics and subsequent resurgence.

Indeed, A(H1N1) pdm09 virus, like other IAVs, has a high mutation rate, which allows it to evolve rapidly and evade host immune responses. This rapid evolution is a key factor in its ability to overcome host barriers and cause recurring epidemics and occasional pandemics [12]. Such evolution may have an impact on the virulence and specificity of the virus–host, influencing epidemiological behavior and perhaps leading to future human pandemics [13]. To achieve this goal, annual surveillance of the influenza virus is crucial in supplying vital information for the annual reformulation of influenza vaccines [14].

The effectiveness of the influenza vaccine is mostly influenced by the significant mutation rate of the influenza virus. Reports indicate effectiveness rates ranging from 30% to 60% against influenza, contingent upon the antigenic compatibility between the vaccine and the circulating strain [15,16]. Most influenza vaccines are designed to target the influenza virus’s hemagglutinin (HA) and neuraminidase (NA) surface glycoproteins or antigens. The pdm09 HA is essential for binding to host cell receptors and promoting the release of viral RNA into the cell, similar to other subtypes [17]. The three distinct domains that make up the receptor-binding domain (RBD) within a globular head domain are the 130-loop (residues 134–138), the 190-helix (residues 188–195), and the 220-loop (residues 221–228). This structure particularly targets host cell sialic acid residues [18,19,20]. The frequency of amino acid changes in influenza indicates that the antigens in the yearly vaccine must be modified consistently to safeguard against current strains. The selection of strains is based on the identification of novel variations and the evaluation of antigenic change using vaccines’ sera and ferret antisera [21]. Since its emergence, A(H1N1) pdm09 has evolved into distinct phylogenetic clades, such as 6B.1 and 5a.1, with key subclades defined by amino acid substitutions like K163Q in clade 6B.1A and D187A/Q189E in 5a.2. Globally detected substitutions in HA (e.g., S183P, S185T) and NA (e.g., V106I) have influenced antigenicity, leading to WHO vaccine updates, such as from A/Michigan/45/2015 (clade 6B.1) to A/Wisconsin/67/2022 (clade 5a.2a.1) for better match against circulating strains [21].

In Saudi Arabia, multiple studies have documented the presence of the influenza A(H1N1) pdm09 virus across different areas; A(H1N1) pdm09 was represented 27.3% in the Western region of Saudi Arabia over the four seasons from October 2015 to 2019 [22]. Similarly, the IAV rate was recorded at 151 (88%) among 778 outpatients presenting with influenza-like symptoms. Of these cases, 43 (5.5%) were identified as A(H1N1) pdm09 in Riyadh, Saudi Arabia, during the 2019/2020 season [23]. In 2020–2022, 21 out of 200 clinical samples (10.5%) from hospitalized children in Riyadh were positive for IAV, with A(H1N1) pdm09 accounting for 71.4% of isolates [24]. Furthermore, we reported that the prevalence of IAV was 28.3% (88 out of 311 samples) among the five seasons investigated (2014–2018 and 2019/2020), with H1N1 strains showing a higher prevalence at 51.2% (45 out of 88 samples) [25]. Similarly, we have recently indicated that from the 363 samples collected, 110 were positive for IAV. Among these positive samples, the A(H1N1) pdm09 strain was the most significant, with a count of 68 (61.8%) during the 2024–2025 season [26]. Although these studies have provided valuable information on the epidemiology of the A(H1N1) pdm09 subtype in Saudi Arabia, the understanding of the molecular epidemiology and genetic diversity of this virus in the nation is still markedly underexplored. Continuous genetic and antigenic monitoring is crucial for identifying mutations that influence antigenicity, especially in regions where data is limited.

In this analysis, we explored the evolution of A(H1N1) pdm09 by examining several available HA and NA sequences from the A(H1N1) pdm09 strain across various geographic regions. We discovered multiple mutation sites within the *HA* and *NA* genes of the A(H1N1) pdm09 virus. It was noted that mutations at various positions resulted in changes in amino acids, which were associated with vaccine strains, and numbers of mismatches were found between the vaccine strains and the circulating influenza A(H1N1) pdm09. We suggest considering these local strains as potential candidates for regional vaccine evaluation in upcoming seasons, pending further antigenic and global surveillance data. Thus, our evolutionary analysis may enhance the selection process for vaccine strain candidates, improve vaccine effectiveness, and contribute to disease management.

## 2. Results

### 2.1. Prevalence of IAV

A total of 380 clinical samples were examined over the 2020–2023 winter seasons (Table 1). These were 14 (11.67%) samples collected from the years 2020–2021, 17 (14.17%) in 2021–2022, and 34 (26.15%) in 2022–2023. Of these samples, IAV was identified in 17.11% of the samples, with A/H3N2 (9.21%) slightly higher than A(H1N1) pdm09 (7.89%). There were 16 males 16 (9.41) and those aged 0–4 represented the majority, with 13 (13.68%) (*p* < 0.05).

Among individuals with IAV, the most frequently reported symptoms were headache (92.31%), nasal congestion (92.31%), cough (84.62%), sore throat, myalgia (83.08%), and fever (75.38%) (Table 1).

Statistical testing demonstrated that children aged 5–14 years had a significantly higher proportion of IAV cases compared with the general population, whereas the distribution within the 0–14-year age group was significantly uneven (*p* < 0.05; Table 2).

### 2.2. Sequence Analysis of the HA and NA Genes of A(H1N1) pdm09 Isolates

#### 2.2.1. Homology Analysis *HA* and *NA* Genes of A(H1N1) pdm09 Isolates

A total of 92 (*HA* and *NA* genes) of A(H1N1) pdm09 strains were retrieved from the GenBank and GISAID databases; these strains illustrate a range of viral clades, countries, and years. The nucleotide sequence similarities of the study isolates were compared to the prototypic A(H1N1) pdm09 reference A/California/7/2009; the sequence analysis revealed that nucleotide identity ranged from 98.2 to 98.9% (HA), while the NA identity was found to be between 97.2 and 98.3%.

#### 2.2.2. Analysis of Amino Acid Sequences of the *HA* Gene in A(H1N1) pdm09 Isolates

**Figure 1:**
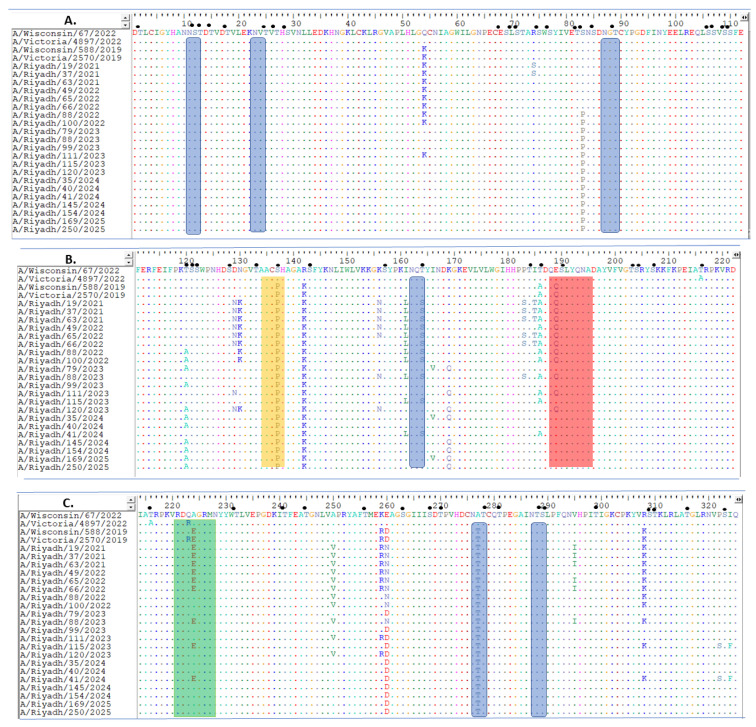
Alignment of deduced amino acid sequences of the HA1 domain of the *HA* gene of A(H1N1) pdm09 isolates. The amino acid positions corresponded to amino acid positions relative to the sequences of current vaccine strains of A/Wisconsin/67/2022). (**A**) Amino acid residues from 1 to 112, (**B**) from 113 to 222, and (**C**) from 214 to 325. Dots indicate identical residues and different residues are shown in a single-letter code. Different amino acids are marked in different colors, and identical residues are presented by dots. 130-loop (residues 134–138) is represented by the enclosed orange rectangle, the 190-helix (residues 188–195) is represented by the enclosed red rectangle, and the 220-loop (residues 221–228) is represented by the enclosed green rectangle. Predicted N-glycosylation sites are enclosed in blue rectangles. Small, filled circles correspond to predicted O-glycosylation sites. The amino acid sequences for A(H1N1) pdm09 isolates from 2024 and 2025 has been published [26].

In comparison to the vaccine strains (A/Victoria/4897/2022, and A/Wisconsin/67/2022), a total of 53-point mutations have been identified, among which 24 have resulted in changes to their corresponding amino acids. Of these amino acid residues, 14 were permanently changed in the majority of isolates: Q54K, S137P, R142K, I161L, T164S, T168A, T186A, E189Q, A224E, A250V, K259R, E260D/N, A277T, and R308K (Figure 1A–C).

In a receptor-binding domain (RBD), we found several antigenicity-related homologous sequences, including the 130-loop (residues 134–138), the 190-helix (residues 188–195), and the 220-loop (residues 221–228), when we compared the *HA* gene of the Riyadh isolates to the vaccine strains A/Wisconsin/588/2019, A/Victoria/2570/2019, (2021–2022 and 2022–2023), A/Victoria/4897/2022, and A/Wisconsin/67/2022, recommended for the 2023–2024, 2024–2025, and 2025–2026 winter seasons (Figure 1B,C). Comparison with vaccine strains (A/Victoria/4897/2022) revealed similar patterns, with 20–25 amino acid changes per season, including the persistent S137P within the 130-loop in Riyadh isolates that was absent in all vaccine strains (Figure 1B).

#### 2.2.3. Sequence Analysis of the *NA* Gene of A(H1N1) pdm09 Isolates

**Figure 2:**
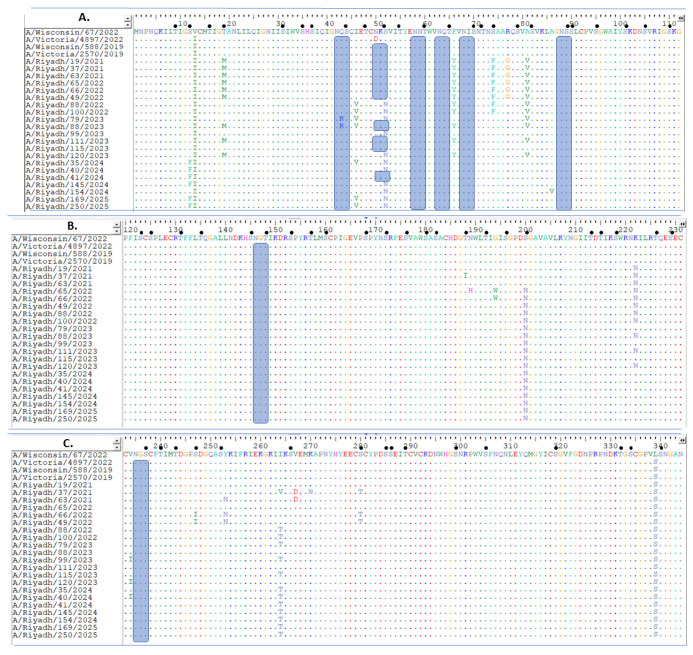

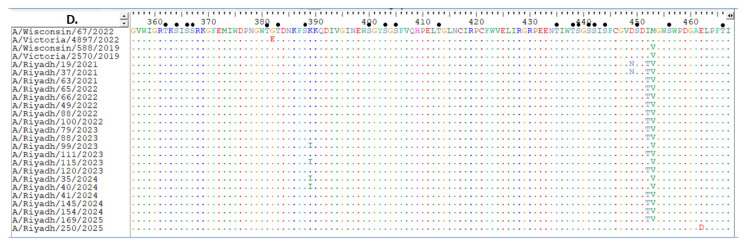
Alignment of deduced amino acid sequences of the *NA* gene of A(H1N1) pdm09 isolates. The amino acid positions corresponded to amino acid positions relative to the sequences of current vaccine strains of A(H1N1) pdm09 (A/Wisconsin/67/2022). (**A**) Amino acid residues from 1 to 112, (**B**) from 120 to 231, (**C**) from 238 to 344, and (**D**) from 356 to 467. Dots indicate identical residues, and different residues are shown in a single-letter code. Different amino acids are marked in different colors; identical residues are presented by dots. Predicted N-glycosylation sites are enclosed in blue rectangles. Small, filled circles correspond to predicted O-glycosylation sites. The amino acid sequences for A(H1N1) pdm09 isolates from 2024 and 2025 have been published [26].

Likewise, the complete nucleotide sequence alignment of the *NA* gene of the Riyadh isolates revealed in this study was conducted alongside the corresponding sequences from international and vaccine strains. A comparison with the vaccine strain (A/Wisconsin/67/2022) *NA* gene indicated 24 mutations (1.71), with 16 resulting in modifications to the corresponding amino acids. Of these modified amino acid residues, 11 were identified as altered in most isolates: V13I, T19M, F66Y, S74V, A81V, S200N, K222N, L399S, I452T, M453V, and N469K (Figure 2A–D).

### 2.3. N- and O-Linked Glycosylation Site Analysis of A(H1N1) pdm09 Isolates

The HA1 domain of the A(H1N1) pdm09 isolates’ HA proteins possesses several O-glycosylation sites (60). On the other hand, the Riyadh isolates showed a minor reduction in the number of N-glycosylation sites (6), which were likewise present in the vaccine strain (A/Wisconsin/67/2022) and were 110(NST), 87(NGT), 162(NQT), 276(NAT), 287(NTS), and 481(NGT), (Figure 1A–C). The NA protein from the Riyadh isolates was found to have O-glycosylation sites (64). Conversely, six N-glycosylation sites were found in the Riyadh isolates and the vaccine strain (A/Wisconsin/67/2022) at positions 42(NQS), 50(NKS), 58(NNT), 63(NQT), 146(NGT), and 235 (NGT), (Figure 2A–D).

### 2.4. Phylogenetic Analysis

**Figure 3:**
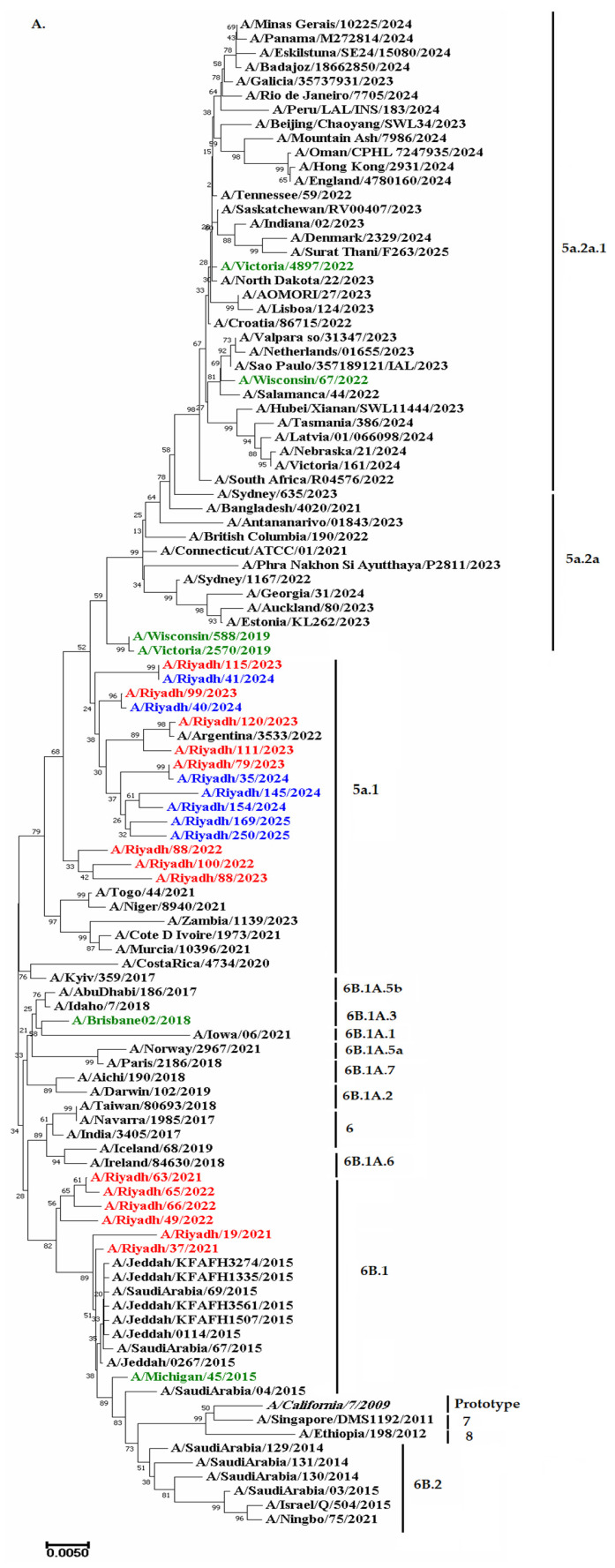

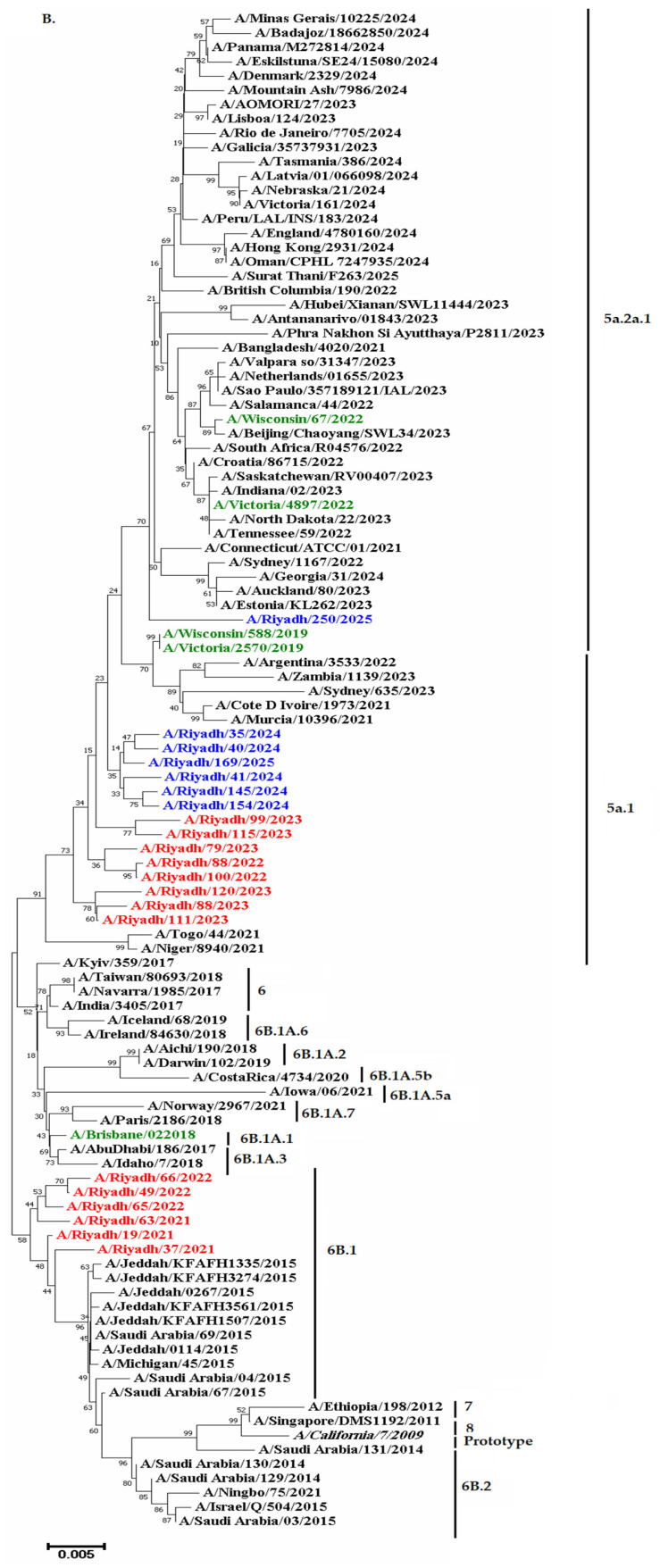
Phylograms of A(H1N1) pdm09 isolates; (**A**) *HA* gene and (**B**) *NA* gene. The Neighbor-Joining method of the MEGA 11 (v.11, Pennsylvania State University, University Park, PA, USA) program was used to determine a phylogenetic tree (cladogram) based on nucleotide sequences. The numbers at the internal nodes of the tree represent the bootstrap values of 1000 replicates. Only values exceeding 70% are shown. Riyadh isolates identified in the current study are shown in red color. Riyadh isolates from 2024 and 2025 have been published [26], shown in blue. Those in green represent WHO vaccine reference strains for the Southern Hemisphere. Reference and prototype strains are denoted by italic font.

Phylogenetic trees constructed from *HA* and *NA* gene sequences were utilized to assess the evolutionary relationships between Riyadh A(H1N1) pdm09 isolates (*n* = 14) and reference strains (*n* = 92), as well as to compare their genetic similarity to contemporary viruses circulating in other countries. Most of the Riyadh isolates (*n* = 8) from the three seasons (2020–2023), in addition to all of Riyadh isolates (*n* = 7) from the 2024/25 season, were categorized into (5a.1), indicating the highest genetic similarity to the A/Argentina/3533/2022 strain, with a sequence homology of 97%, whereas the remaining A(H1N1) pdm09 isolates (*n* = 6) were found in Cluster 6B.1 (Figure 3A,B).

## 3. Discussion

Influenza is a major public health issue, characterized as an acute respiratory infection caused by the influenza virus, which occurs annually in seasonal outbreaks peaking during the winter and spring months [27]. We have recently examined the molecular epidemiology and genetic changes and analyzed the correspondence with vaccine strains of the influenza A(H1N1) pdm09 subtype that is circulating in Riyadh, Saudi Arabia, in the 2024–2025 season [26]. Despite this, there is a lack of knowledge concerning the circulating strain of the A(H1N1) pdm09 subtype in Riyadh during the period of 2020–2023. Regular genetic and antigenic monitoring is important for tracking mutations that may influence antigenicity, particularly in areas with insufficient data. Through the examination of several HA and NA sequences obtained from the A(H1N1) pdm09 strain in various geographical locations, the current study sought to clarify the evolution of A(H1N1) pdm09. Our research identified many mutation sites in the A(H1N1) pdm09 virus’s *HA* and *NA* genomes. Several differences between the vaccine strains and the circulating influenza A(H1N1) pdm09 were found, and it was noted that mutations at different places caused changes in amino acid sequences that related to vaccination strains. We support taking these strains into account as possible vaccine candidates for the impending influenza season. As a result, our evolutionary study could improve the process of choosing potential vaccine strains, increasing the effectiveness of vaccines.

The total prevalence of IAV in the current research was 65 (17.11%), and 7.89% (30/380) of them had the A(H1N1) pdm09 virus. Most of them were 0–4 years old (13.68%) and male (16, 9.41%) over three winter seasons in Riyadh from 2020 to 2023. Our observed lower IAV detection rates in 2020–2021 (11.67% of samples collected) and gradual increase toward 2022–2023 (26.15%) align with global trends during the COVID-19 pandemic. Non-pharmaceutical interventions suppressed influenza circulation worldwide, with reports of substantial declines (often exceeding 95% in detections in Europe and globally) during 2020–2021, reduced severity in remaining cases, and resurgence by 2022 as restrictions eased [10,11]. This likely contributed to the limited A(H1N1) pdm09 diversity and sample availability early in our study period, followed by increased circulation and the observed genetic changes (e.g., persistent S137P).

Our study found that among individuals infected with IAV, the most frequently reported symptoms were headache (92.31%) and nasal congestion (92.31%), followed by cough (84.62%), sore throat, myalgia (83.08%), and fever (75.38%). These findings are consistent with the classic influenza syndrome, which typically has a sudden onset and is characterized by fever, headache, cough, sore throat, myalgia, nasal congestion, generalized weakness, and loss of appetite [28].

Vaccination remains the most effective method of preventing influenza infections and associated aftereffects, particularly for high-risk groups such as young children, the elderly, and those with chronic medical conditions [29]. The HA and NA proteins are the main targets of a universal vaccine against the influenza virus. It is crucial to comprehend the evolution and diversity of HA when it comes to influenza immunization. When creating vaccines that target neutralizing reactions to HA and/or NA, antigenic drift must be considered. Although seasonal influenza vaccinations successfully prevent infection by inducing neutralizing antibodies against the HA head, the rapid mutation of viruses poses serious challenges to this strategy. The WHO arranges and conducts vaccine strain consultation workshops twice a year for IAV and IBV vaccines in order to choose human immunization strains for the Northern or Southern Hemispheres [30].

The vaccine strain A/Wisconsin/67/2022 was used to assess the effectiveness of the current vaccination against the circulating A(H1N1) pdm09 isolates from Riyadh. Seven amino acid changes were consistent across most isolates: S137P, R142K, I161L, T164S, T168A, I401V, E189Q, A224E, A250V, A260N, A277T, R308K, D356E, Q389L, V418I, H451N, and D506E. These changes, notably in the HA1 domain, are crucial because they occur in antigenic areas such as the 120-loop, 150-loop, 160-loop, and 190-helix, respectively. Changes in these regions may potentially alter the virus’s antigenicity and contribute to immune evasion. All the isolates from Riyadh, however, showed a permanent alteration in the 130-loop (S137P substitution), unlike the vaccine strains. This substitution is hypothesized to potentially enhance immune evasion and viral persistence; however, functional validation through hemagglutination inhibition (HAI) assays is required to confirm its impact on antigenicity [17,18,19,20,31].

The whole nucleotide sequence alignment of the *NA* gene from Riyadh isolates shows significant genetic heterogeneity as compared to global and vaccine strains. V13I, T19M, F66Y, S74V, A81V, S200N, L399S, I452T, M453V, and N469K were among the 24-point mutation sites (1.71%) that resulted in amino acid changes and 10 permanent substitutions as compared to the consensus sequence strain (A/Wisconsin/67/2022). In our recent study conducted by Abdulgader et al. (2025) in Riyadh, Saudi Arabia, they identified two unique amino acid substitutions (S12F and S52N) that were reported in our strains but not found in the international strains [26]. A previous study has shown that some mutations in the enzymatic active region, such D151G, may reduce neuraminidase activity, which would affect the enzyme’s ability to release viruses and cleave sialic acid residues [32]. These findings are consistent with the alterations in the Riyadh isolates, suggesting possible effects on viral fitness and immunological evasion.

In this analysis, identifying the appropriate reference strains was vital to contextualizing the evolutionary history of the present-day A(H1N1) pdm09 isolates. A/California/7/2009 served as the baseline reference for our mutational and phylogenetic studies. This is supported by decades of global sequencing data from GISAID and GenBank [33]. It is common practice in pandemic flu research to use this progenitor strain as a global reference, which aids in monitoring changes over time and provides comparisons to clades across multiple countries [24,25].

In terms of O-linked and N-linked glycosylation sites, the glycosylation patterns of the HA and NA proteins in the Riyadh isolates were similar to those of the vaccine strain (A/Wisconsin/67/2022). Glycosylation of the influenza’s HA and NA provides crucial tactics for immune evasion and viral fitness in a host population. Glycosylation in HA is necessary for the protein’s folding and stability [34,35], and, in some situations, has a major impact on receptor binding and cleavage of the precursor HA0 protein, which influences the virus’s virulence and antigenicity [36]. Furthermore, glycosylation on the HA globular head domain may physically protect the antigenic sites, inhibiting antibody detection and allowing the virus to evade antibody-mediated neutralization [37,38,39]. 

Increased O-glycosylation is linked to decreased antibody binding, permitting increased viral production and creating a glycan shield that shields the virus from neutralizing antibodies [40,41,42,43,44,45].

The phylogenetic analysis revealed that all of the Riyadh isolates (*n* = 7) from the 2024/25 season and the majority of the isolates (*n* = 8) from the three seasons (2020–2023) were classified into 5a.1, indicating the highest genetic similarity to A/Argentina/3533/2022, A/Togo/44/2021, and A/Niger/8940/2021. The circulating strains in Riyadh during the epidemic years of 2020–2021 and 2021–2022 were found to belong to the same clade [24]. However, a different study showed that the clade of the circulating A/H1N1 in Riyadh during the 2021–2022 pandemic year was 5a.2a [46]. These variations may be caused by the fact that several virus introductions have a substantial impact on local epidemic evolution, allowing different clades of the same subtype to circulate at the same time [33,47]. Global epidemiological trends are mostly shaped by the spread of viruses from regions with longer-lasting influenza transmission, especially in East and South-East Asia [48,49,50].

The current study provides new information about the epidemiology, genetic evolution, and antigenic variety of A(H1N1) pdm09 in Riyadh, Saudi Arabia, throughout the 2020–2023 epidemic seasons. Unlike earlier studies, which focused mainly on prevalence and seasonal fluctuations [22,23,51], our work uses extensive genomic and phylogenetic analysis to uncover the breadth of viral evolution and immune evasion mechanisms. Compared to prior studies [24,51] and WHO recommendations for influenza vaccine composition (e.g., A/Wisconsin/67/2022), this article’s novelty is identifying the persistent S137P substitution in Riyadh strains absent in vaccine compositions, alongside increased O-glycosylation potentially enhancing local immune evasion. Unlike global reports focusing on clades 6B.1/5a.2, we highlight 5a.1 dominance in Riyadh, providing region-specific data for vaccine updates in understudied Middle Eastern contexts.

This study’s main limitation is the small sample size (14 sequenced isolates), which may limit generalizability for broader virus evolution inferences. The 14 isolates represent successfully sequenced strains from 30 positive A(H1N1) pdm09 detections out of 380 samples, constrained by challenges in clinical sample quality and amplification success during the COVID-19 period. However, this sample provides valuable insights into local circulation in an understudied region, complementing global data. Moreover, it does not identify the reason why infections repeat. More thorough research with bigger sample numbers dispersed throughout Saudi Arabia’s many regions, including the major cities, during subsequent epidemic seasons, is required to provide a more thorough understanding of the circulation patterns of A(H1N1) pdm09. Future studies should also include HAI assays to validate the functional impact of key mutations, such as S137P, on antigenicity and vaccine effectiveness.

## 4. Materials and Methods

### 4.1. Study Population, Consent Forms, and Ethics

This analysis was performed on 380 nasopharyngeal aspirates (NPAs) sourced from patients with influenza-like illness (ILI) [25], characterized by symptoms like fever, cough, dyspnea, and rhinorrhea, during the winter timeframe (November to March of the next year) from 2020 to 2023 in Riyadh, Saudi Arabia. The demographics and clinical characteristics of the patients who were included in the study were collected from their medical records. This encompassed age, gender, admission status (inpatient or outpatient), and symptoms. The study was conducted in accordance with the Declaration of Helsinki, and approved by the Research Ethics Committee, King Saud University (KSU) in Riyadh, Saudi Arabia (Institutional Review Board No. 22/0957/IRB). Following collection, two milliliters of viral minimal essential medium (MEM) transport medium (Gibco, Invitrogen, Grand Island, NY, USA) were added to the samples. After that, they were shipped in a refrigerator to the KSU College of Science’s Virology Research Laboratory, where they were kept at −80 °C until additional examination.

### 4.2. Identification and Determination of Influenza Subtype

The QIAamp Viral RNA Extraction Kit (Qiagen, Hilden, Germany) was used in accordance with the manufacturer’s instructions to extract RNA from clinical samples obtained during the research periods. IAV was detected using the universal primer as described in our recent study [26], and One-Step Ahead RT-PCR Kit with Taq High Fidelity DNA Polymerase (Qiagen, Hilden, Germany).

Additionally, using the same amplification kit and two sets of overlapping primers (all primers were described in our recent study [26], IAV-positive samples were further examined for genetic subtyping, including the *HA* and *NA* genes. GeneAmp 9700 thermal cycler (Applied Biosystems, Foster City, CA, USA) was used for all RT-PCR investigations, and the cycling conditions were as follows: For thirty minutes, reverse transcription was carried out at 50 °C. A first denaturation phase at 95 °C for 15 min came next. The PCR products were examined under UV light using an agarose gel stained with 1% ethidium bromide and compared to a 100 bp Plus DNA ladder (Qiagen, Hilden, Germany).

### 4.3. Amplification and Sequencing of HA and NA Genes

Using the same kit and primer sets, a second round of RT-PCR was performed on the samples that showed positive typing results to sequence the full-length *HA* and *NA* genes in IAV-positive samples (Table S1). The resulting amplified *HA* (1701 bases) and *NA* (1410 bases) gene segments were sequenced in both directions by Macrogen Inc. (Seoul, Republic of Korea). The raw sequence data were modified using the BioEdit program, version 7.0 (Ibis Biosciences, Carlsbad, CA, USA), and then assembled using the Edit sequence tool of the MegAlign program, Lasergene software, version 3.18 (DNAStar, Madison, WI, USA). Based on their sequence heterogeneity, only 14 sequences were chosen for phylogenetic and sequencing analysis. Only a small portion of the 90 positive Influenza A(H1N1) pdm09 strains are represented by the 14 samples that were examined. However, they emphasized that these 14 strains were the ones that successfully underwent both *HA* and *NA* gene amplification and sequencing. This procedure is often the bottleneck in molecular surveillance studies, particularly when working with clinical samples that may have varied viral loads and RNA integrity. The ultimate sequences in this investigation have been archived in GenBank, with accession numbers ranging from PX487272 to PX487285 for the *HA* gene and from PX487296 to PX487309 for the *NA* gene.

### 4.4. Sequence and Phylogenetic Analysis

Ninety-two local and worldwide circulating strains of A(H1N1) pdm09 were obtained from the Gene Bank website and the GISAID database, and the sequences of the *HA* and *NA* genes were aligned using the Clustal W method. To illustrate evolutionary divergence from the pandemic origin, alignments were made against the prototypic A(H1N1) pdm09 reference A/California/7/2009 (GenBank: NC_026433 for HA; NC_026434 for NA). Lasergene software (DNAStar Inc., Madison, WI, USA) and the EditSeq and MegAlign programs were used for divergence analysis, mutation site identification, and amino acid change prediction.

We compared our sequences with the WHO-recommended vaccine strains A/Wisconsin/588/2019 (cell-based) and A/Victoria/2570/2019 (egg-based), which were used for the 2021–2022 and 2022–2023 winter seasons; A/Victoria/4897/2022, recommended for the 2022–2023 season; and A/Wisconsin/67/2022, recommended for the 2023–2024, 2024–2025, and 2025–2026 winter seasons. These comparisons were performed to assess current and near-future vaccine relevance in the context of the ongoing circulation of related clades. Additionally, comparisons with earlier WHO-recommended strains have now been included where appropriate. EditSeq, MegAlign, and Lasergene software (DNAStar Inc., Madison, WI, USA) were used to calculate the genetic distances of nucleotide and amino acid sequences between Jiaxing influenza A(H1N1) isolates and the vaccine strain using the WHO-recommended 2020–2023 Northern Hemisphere A(H1N1) pdm09 vaccine virus A/Wisconsin/67/2022 as a consensus sequence. Next, a phylogenetic tree was constructed using the Neighbor-Joining technique, with a bootstrap analysis set to 1000 repetitions. A total of 14 sequences from this study and 85 A(H1N1) pdm09virus sequences from the GISAID database and the Gene Bank website that came from various nations were used to build the phylogenetic tree (Table S1).

NetNGlyc 1.0 [52], and NetOGlyc 3.1 [53] were used to determine heterogeneity in N-glycosylation (Asn-X-Ser/Thr, where X stands for any amino acid except proline) and O-glycosylation sites (Ser or Thr) within the HA and NA proteins of A(H1N1) pdm09. The neighbor-joining method in MEGA 11 (v.11, Pennsylvania State University, University Park, PA, USA) was used to carry out the phylogenetic analysis, with 1000 bootstrap replications. For clarity, bootstrap values over 70% were shown on the main tree branches.

### 4.5. Statistical Analysis

IBM SPSS Statistics v26.0 (IBM Corp., Armonk, NY, USA) was used for statistical analysis. Fisher’s exact test was used to assess categorical variables, and Bonferroni correction was applied to post hoc Z-tests. Statistical significance was defined as a significance level of *p* < 0.05.

## 5. Conclusions

This study provides thorough molecular monitoring of IAV circulation in Riyadh, Saudi Arabia from 2020 to 2023. IAV was identified in 17.11% of the samples, with A/H3N2 (9.21%) slightly higher than A(H1N1) pdm09 (7.89%). The majority of participants were 0–4 years old (13.68%) and male (16, 9.41%), with samples taken over three winter seasons in Riyadh from 2020 to 2023, in Riyadh, Saudi Arabia. Using molecular surveillance, we found genetic evidence of potential antigenic mismatch between the current vaccination strain (A/Wisconsin/67/2022) and the strains that are currently circulating locally. Interestingly, at position S137P in the RBD of the HA protein, all local isolates exhibit a constant amino acid substitution. Such mutation is absent in the vaccine strain and is characteristic of improved immune evasion and viral persistence. The findings show the potential advantages of bivalent vaccine techniques for wider protection and emphasize the necessity of ongoing genomic surveillance to identify new variations that may undermine preventive measures. The data provided here is crucial for guiding the yearly selection of vaccination candidates, especially for Saudi Arabia. Furthermore, the work makes a substantial contribution to worldwide influenza surveillance initiatives and the development of future vaccines that are more potent. These genetic observations highlight the need for antigenic studies, such as HAI assays, to confirm mismatch and immune evasion. It is suggested that these locally circulating strains be used as possible vaccine candidates to improve protection against the predominant variants in this region.

## Figures and Tables

**Table 1:** Sample distribution across epidemic seasons (winters 2020–2023), gender, and age groups.

		No. of Samples *n* (%)	Positive for IAV *n* (%)	Positive for	
				A(H1N1) pdm09 *n* (%)	A/H3N2 *n* (%)
Total		380	65 (17.11)	30 (7.89)	35 (9.21)
Season	2020/21	120 (31.57)	14 (11.67)	6 (5.00)	8 (6.67)
	2021/22	130 (34.21)	17(14.17)	6 (4.62)	11 (8.46)
	2022/23	130 (34.21)	34 (26.15)	18 (13.84)	16 (12.30)
Gender	Male	170 (44.74)	44 (25.88) ^a^	16 (9.41) ^a^	28 (16.47) ^a^
	Female	210 (55.26)	21 (10.00)	14 (6.67)	7 (3.33)
Age in years	0–4	95 (20.00)	27 (28.42) ^b^	13 (13.68) ^b^	14 (14.75) ^b^
	5–14	110 (28.95)	19 (17.27)	7 (6.35)	12 (10.91)
	15–64	120 (31.57)	9 (7.50)	4 (3.33)	5 (4.16)
	≥65	55 (14.48)	10 (18.18)	6 (10.91)	4 (7.27)
Symptoms or sign	Fever >°C		49 (75.38)	21 (70.00)	28 (00.00)
	Cough		55 (84.62)	23 (76.67)	32 (91.43)
	Nasal congestion		60 (92.31)	25 (83.33)	35 (100.00)
	Weakness		40 (61.54)	13 (43.33)	27 (77.14)
	Sore throat		41 (63.08)	12 (40.00)	29 (82.86)
	Headache		60 (92.31)	27 (90.00)	33 (94.83)
	Myalgia		54 (83.08)	21 (70.00)	33 (94.83)

Data are displayed as percentages (%). ^a^ Significantly higher (*p* < 0.05) than males. ^b^ Significantly higher (*p* < 0.05) than age groups 5–14, 15–64, and ≥65 years.

**Table 2:** Sample distribution across epidemic seasons (winters 2020–2023) according to age groups.

	2020/21				2021/22				2022/23			
	No. of Samples *n* (%)	IAV *n* (%)	A(H1N1) pdm09 *n* (%)	A(H3N2) *n* (%)	No. of Samples *n* (%)	IAV *n* (%)	A(H1N1) pdm09 *n* (%)	A(H3N2) *n* (%)	No. of Samples *n* (%)	IAV *n* (%)	A(H1N1) pdm09 *n* (%)	A(H3N2) *n* (%)
Total	120	14 (11.67)	6 (5.00)	8 (6.67)	130	17 (13.07)	6 (4.62)	11 (8.46)	130	34 (26.15)	18 (13.84)	16 (12.31)
0–4 years	32	1 (3.13)	1 (3.13)	0 (0.00)	35	6 (17.14)	2 (5.71)	4 (11.43)	28	20 (71.42) *	10 (35.71) *	10 (35.71) *
5–14 years	35	7 (20.00) *	3 (8.57)	4 (11.43)	40	6 (15.00)	2 (5.00)	4 (10.00)	35	6 (17.14)	2 (5.71)	4 (11.43) *
15–64 years	43	2 (4.65)	1 (2.32)	1 (2.32)	37	3 (8.11)	1 (2.70)	2 (5.41)	40	4 (10.00)	2 (5.00)	2 (5.00)
≥65 years	10	4 (40.00)	1 (10.00)	3 (30.00)	18	2 (11.11)	1 (5.56)	1 (5.56)	27	4 (14.82)	4 (14.82)	0 (0.00)

* Significantly higher (*p* < 0.05) than age groups 5–14, 15–64, and ≥65 years.

## Data Availability

The datasets generated and analyzed during the current study are available from the corresponding author upon reasonable request. The sequence data reported in this study have been deposited in GenBank under accession numbers (*HA* gene: PX487272 to PX487285 and *NA* gene: PX487296 to PX487309).

## Supplementary Materials

The following supporting information can be downloaded at: https://www.mdpi.com/article/10.3390/ijms27031412/s1.
