# Supplementary material for: Molecular Surveillance, Evolution, and Vaccine Strain Match of the HA and NA Genes of 2009 H1N1 Pandemic Virus Circulating in Riyadh, Saudi Arabia
Source: Int J Mol Sci. 2026 Jan 30;27(3):1412. doi: 10.3390/ijms27031412 (PMC12897650; doi:10.3390/ijms27031412)
Supplement: Supplementary file 1 [file ijms-27-01412-s001.zip › ijms-4078945-supplementary.pdf]

Table S1: List of H1N1 strains included in sequence and phylogenetic analysis.

No.	Strain	Origin	GenBank accession No.			
			H1	N1	Clade	Note
1.	A/Michigan/45/2015	USA	MK622940	MK622934	6B.1	Vaccine strain
2.	A/Brisbane/02/2018	Australia	EPI1504919	EPI1504919	6B.1A.1	Vaccine strain
3.	A/India/3405/2017	India	EPI1161973	EPI1161972	6	
4.	A/Taiwan/80693/2018	Taiwan	EPI1281129	EPI1281128	6	
5.	A/Navarra/1985/2017	Spain	EPI1032468	EPI1281128	6B.1A.1	
6.	A/Aichi/190/2018	Japan	EPI1267660	EPI1267659	6B.1A.2	
7.	A/Darwin/102/2019	Australia	EPI1435917	EPI1435916	6B.1A.2	
8.	A/AbuDhabi/186/2017	UAE	EPI1180204	EPI1180203	6B.1A.3	
9.	A/Idaho/7/2018	USA	EPI1206974	EPI1206973	6B.1A.3	
10.	A/California/7/2009	USA	NC_026433	NC_026434	Protype 4	Vaccine strain
11.	A/Sydney/1167/2022	Australia	EPI2152082	EPI2152081	6B.1A.5a.2	
12.	A/Kyiv/359/2017	Ukraine	EPI1164245	EPI1164243	6B.1A.5b	
13.	A/CostaRica/4734/2020	Costa Rica	EPI1805661	EPI1805660	6B.1A.5b	
14.	A/Ireland/84630/2018	Ireland	EPI1354717	EPI1354718	6B.1A.6	
15.	A/Iceland/68/2019	Iceland	EPI1506821	EPI1506822	6B.1A.6	
16.	A/Paris/2186/2018	France	EPI1313521	EPI1313520	6B.1A.7	
17.	A/Norway/2967/2021	Norway	EPI1882571	EPI1882570	6B.1A.7	
18.	A/Israel/Q/504/2015	Israel	EPI697729	EPI697730	6B.2	
19.	A/Ningbo/75/2021	China	EPI1858137	EPI1858139	6B.2	
20.	A/Singapore/DMS1192/2011	Singapore	EPI634252	EPI634680	7	
21.	A/Ethiopia/198/2012	Ethiopia	EPI386042	EPI386041	8	
22.	A/SaudiArabia/129/2014	Saudi Arabi	MK246085	MK228959		
23.	A/SaudiArabia/130/2014	Saudi Arabi	MK246086	MK228960		

24.	A/SaudiArabia/131/2014	Saudi Arabi	MK246087	MK228961		
25.	A/Saudi Arabia/67/2015	Saudi Arabi	MK246043	MK228917		
26.	A/Saudi Arabia/69/2015	Saudi Arabi	MK246045	MK228919		
27.	A/Saudi Arabia/03/2015	Saudi Arabi	MK246015	MK228889		
28.	A/Saudi Arabia/04/2015	Saudi Arabi	MK246016	MK228890		
29.	A/Jeddah/KFAFH3561/2015	Saudi Arabi	MF768811	MF768813		
30.	A/Jeddah/KFAFH3274/2015	Saudi Arabi	MF768755	MF768757		
31.	A/Jeddah/KFAFH1507/2015	Saudi Arabi	MF768779	MF768781		
32.	A/Jeddah/KFAFH1335/2015	Saudi Arabi	MF768731	MF768733		
33.	A/Jeddah/0267/2015	Saudi Arabi	MF768787	MF768789		
34.	A/Jeddah/0114/2015	Saudi Arabi	MF768595	MF768597		
35.	A/Wisconsin/67/2022-cell based	USA	EPI2224978	EPI2224977	5a.2a.1	Vaccine strain
36.	A/Victoria/4897/2022-gg based	Australia	EPI2319193	EPI2319192	5a.2a.1	Vaccine strain
37.	A/Wisconsin/588/2019-cell based	USA	EPI1661231	EPI1661230	5a.2	Vaccine strain
38.	A/Victoria/2570/2019e-gg based	Australia	EPI1718610	EPI1718609	5a.2	Vaccine strain
39.	A/Iowa/06/2021	USA	EPI1941482	EPI1941481	6B.1A	
40.	A/Togo/44/2021	Togo	EPI1987609	EPI1987608	5a.1	
41.	A/Niger/8940/2021	Niger	EPI1951469	EPI1951468	5a.1	
42.	A/Cote_D_Ivoire/1973/2021	Cote d'Ivoir	EPI1885593	EPI1885592	5a.1	
43.	A/Murcia/10396/2021	Spain	EPI1987658	EPI1993865	5a.1	
44.	A/Argentina/3533/2022	Argentina	EPI2316317	EPI2316318	5a.1	
45.	A/Zambia/1139/2023	Zambia	EPI2901545	EPI2901543	5a.1	
46.	A/Connecticut/ATCC/01/2021	USA	EPI2754893	EPI2754892	5a.2a	
47.	A/Croatia/86715/2022	Croatia	EPI2134571	EPI2134572	5a.2a.1	

48.	A/Sao_Paulo/357189121/IAL/2023	Brazil	EPI2717449	EPI2717447	5a.2a.1	
49.	A/Indiana/02/2023	USA	EPI2619801	EPI2619795	5a.2a.1	
50.	A/Mountain_Ash/7986/2024	United Kingdom	EPI3596992	EPI3596994	5a.2a.1	
51.	A/Tasmania/386/2024	Australia	EPI3545286	EPI3545284	5a.2a.1	
52.	A/Denmark/2329/2024	Denmark	EPI3626324	EPI3626323	5a.2a.1	
53.	A/Georgia/31/2024	USA	EPI3246388	EPI3246387	5a.2a	
54.	A/Auckland/80/2023	New Zealand	EPI2927397	EPI2927388	5a.2a	
55.	A/British_Columbia/190/2022	Canada	EPI2127424	EPI2127423	5a.2a	
56.	A/Estonia/KL262/2023	Estonia	EPI2639656	EPI2639657	5a.2a	
57.	A/Minas_Gerais/10225/2024	Brazil	EPI3573271	EPI3573269	5a.2a.1	
58.	A/Valparaso/31347/2023	Chile	EPI3179863	EPI3179862	5a.2a.1	
59.	A/Rio_de_Janeiro/7705/2024	Brazil	EPI3401250	EPI3401248	5a.2a.1	
60.	A/Galicia/35737931/2023	Spain	EPI3069030	EPI3069028	5a.2a.1	
61.	A/Netherlands/01655/2023	Netherlands	EPI2982402	EPI2982401	5a.2a.1	
62.	A/South_Africa/R04576/2022	South Africa	EPI2122017	EPI2122018	5a.2a.1	
63.	A/Hong_Kong/2931/2024	China	EPI3743850	EPI3743849	5a.2a.1	
64.	A/England/4780160/2024	United Kingdom	EPI3716712	EPI3716711	5a.2a.1	
65.	A/Oman/CPHL_7247935/2024	Oman	EPI3736312	EPI3736311	5a.2a.1	
66.	A/Nebraska/21/2024	USA	EPI3432381	EPI3432380	5a.2a.1	
67.	A/Victoria/161/2024	Australia	EPI3236043	EPI3236042	5a.2a.1	
68.	A/Latvia/01/066098/2024	Latvia	EPI3104025	EPI3104024	5a.2a.1	
69.	A/Beijing/Chaoyang/SWL34/2023	China	EPI2669349	EPI2669348	5a.2a.1	
70.	A/North_Dakota/22/2023	USA	EPI2499505	EPI2499500	5a.2a.1	
71.	A/Bangladesh/4020/2021	Bangladesh	EPI1968732	EPI1968731	5a.2a	

72.	A/Salamanca/44/2022	Spain	EPI2542034	EPI2550076	5a.2a.1	
73.	A/Sydney/635/2023	Australia	EPI2719464	EPI2719460	5a.2a.1	
74.	A/Saskatchewan/RV00407/2023	Canada	EPI2555710	EPI2555709	5a.2a.1	
75.	A/Hubei/Xianan/SWL11444/2023	China	EPI2959682	EPI2959677	5a.2a.1	
76.	A/Phra_Nakhon_Si_Ayutthaya/P2811/2023	Thailand	EPI2713317	EPI2713316	5a.2a	
77.	A/AOMORI/27/2023	Japan	EPI2977131	EPI2977130	5a.2a.1	
78.	A/Peru/LAL/INS/183/2024	Peru	EPI3763395	EPI3763394	5a.2a.1	
79.	A/Antananarivo/01843/2023	Madagascarr	EPI2745337	EPI2762775	5a.2a	
80.	A/Surat_Thani/F263/2025	Thailand	EPI3863330	EPI3863329	5a.2a.1	
81.	A/Tennessee/59/2022	USA	EPI2349367	EPI2349366	5a.2a.1	
82.	A/Lisboa/124/2023	Portugal	EPI2899921	EPI2899920	5a.2a.1	
83.	A/Panama/M272814/2024	Panama	EPI3107920	EPI3107922	5a.2a.1	
84.	A/Eskilstuna/SE24/15080/2024	Sweden	EPI3669587	EPI3669586	5a.2a.1	
85.	A/Badajoz/18662850/2024	Spain	EPI3735570	EPI3735569	5a.2a.1	
86.	A/Riyadh/35/2024	Saudi Arabia	PV652976	PV653584	5a.1	
87.	A/Riyadh/40/2024	Saudi Arabia	PV652977	PV653585	5a.1	
88.	A/Riyadh/41/2024	Saudi Arabia	PV652978	PV653586	5a.1	
89.	A/Riyadh/145/2024	Saudi Arabia	PV652979	PV653587	5a.1	
90.	A/Riyadh/154/2024	Saudi Arabia	PV652980	PV653588	5a.1	
91.	A/Riyadh/169/2025	Saudi Arabia	PV652981	PV653589	5a.1	
92.	A/Riyadh/250/2025	Saudi Arabia	PV652982	PV653590	5a.1	
